# Response to “Treatment compliance and effectiveness in complex PTSD patients with co-morbid personality disorder undergoing stabilizing cognitive behavioral group treatment: a preliminary study” – authors’ reply

**DOI:** 10.3402/ejpt.v5.23792

**Published:** 2014-02-05

**Authors:** Ethy Dorrepaal, Kathleen Thomaes, Johannes H. Smit, Dick J. Veltman, Adriaan W. Hoogendoorn, Anton J. L. M. van Balkom, Nel Draijer

**Affiliations:** 1 Department of Psychiatry, VU University Medical Center Amsterdam, The Netherlands, EMGO Institute, VU University Medical Center, Amsterdam, The Netherlands, PsyQ, Parnassiagroep, The Hague, The Netherlands. Email: e.dorrepaal@psyq.nl; 2 GGZ inGeest, Amsterdam, The Netherlands, Department of Psychiatry, VU University Medical Center, Amsterdam, The Netherlands. Email: k.thomaes@vumc.nl; 3 GGZ inGeest, Amsterdam, The Netherlands, Department of Psychiatry, VU University Medical Center, Amsterdam, The Netherlands, EMGO Institute, VU University Medical Center, Amsterdam, The Netherlands; 4 GGZ inGeest, Amsterdam, The Netherlands Department of Psychiatry, VU University Medical Center, Amsterdam, The Netherlands; 5 GGZ inGeest, Amsterdam, The Netherlands, Department of Psychiatry, VU University Medical Center, Amsterdam, The Netherlands, EMGO Institute, VU University Medical Center, Amsterdam, The Netherlands

In a response to our article “Treatment compliance and effectiveness in complex PTSD patients with personality disorders (…)” (Dorrepaal et al., 2013), Dr. A de Jongh and Dr. E ten Broeke express their concern “that conclusions on data derived from an analysis of a sample of complex PTSD patients previously published in the Journal of Psychosomatics and Psychotherapy (Dorrepaal, et al., 2012), could prematurely encourage clinicians, as well as investigators, to offer their patients similar stabilization programs in affiliated mental health institutions.” In this reply, we would like to explain what we actually encourage clinicians and researchers to do, and why: 1) stabilize when PTSD patients drop-out from, or cannot be included in, first-line treatments due to complexity and comorbidity; 2) conduct treatment effect studies on complex PTSD patients with comorbid severe personality disorder(s) (PD) before generalizing the effectiveness of first-line treatments to this understudied group; and 3) use stabilization treatment to prepare complex PTSD patients with comorbid PD for first-line treatments and as such increase the net number of patients profiting from these treatments.

De Jongh and ten Broeke question the conclusions that we drew from our data, citing “while significant superiority on change scores was absent, responder analysis suggested clinical meaningfulness of adding group treatment.” As we reported in the article describing our clinical trial that they referred to (Dorrepaal et al., 2012), our results were ambiguous and were described accordingly. The authors appear to have misread our conclusions as being based on an analysis of patients completing treatment only, whereas they were based on intention-to-treat (ITT) analyses as well as analyses of completers (Dorrepaal et al., 2012, tables 3–4). Change scores were significant at trend level only, probably because we based our power calculation on Zlotnick et al.'s (1997) randomized controlled trial. These authors found a very low effect size in their treatment as usual comparison condition (TAU: d=0.1), while TAU in our trial achieved a medium effect size. Had we initially based the power of our study on a medium effect size within TAU, almost double the number of patients would have been required to demonstrate significant results. Alternatively, we could have applied a more powerful one-tailed test to our data, which would have yielded statistically significant results as well. Importantly, the secondary responders’ analysis on PTSD symptoms (ITT; p=0.03) was statistically significant and indicated clinical meaningfulness. Moreover, our results are supported by our neuroimaging study showing improvement in terms of increased selective attention and decreased emotional arousal, associated with normalized activation in the dorsal anterior cingulate cortex and anterior insula after adding stabilizing treatment (Thomaes et al., 2012). Therefore, we do not agree that our carefully phrased conclusion of “clinical meaningfulness” is an overstatement of the effectiveness of the intervention, especially in this population.

We agree with De Jongh and ten Broeke's concern that “the effect of (the experimental treatment) over TAU could be considered as ‘placebo effects’”, since our design did not include a comparison to a gold standard treatment or placebo (Dorrepaal et al., 2012). However, we disagree with their suggestion that the effect of the treatment over TAU may have been caused by spontaneous improvement or symptom fluctuation as these would likely affect both conditions, which is obviously the reason to compare such conditions in a controlled trial. Moreover, regression to the mean cannot explain the difference between conditions in our trial, since high symptomatic patients and low symptomatic patients profited equally from the treatment.

De Jongh and ten Broeke considered the attrition rate to be relatively high (although they misread the attrition rate, which was 16% in the total group of 71 patients described in Dorrepaal et al., 2012, 18% in the subgroup of 38 patients receiving the stabilizing treatment described in Dorrepaal et al., 2013, and 15% in the subgroup of 33 patients receiving TAU). However, our attrition rate in this complex population is similar to, and not higher than, the mean attrition reported previously for various PTSD populations and treatments (Bradley, Greene, Russ, Dutra, & Westen, 2005; Foa, Keane, Friedman, & Cohen, 2009). Moreover, post-hoc, our complex PTSD patients with a PD actually showed much lower drop-out rates (10%), even lower than drop-out rates in specific PD treatments, which range from 25 to 51% (Doering et al., 2010). As we argued in our discussion, PTSD patients without PDs (the less severe, smaller part of our population), showed the highest drop-out rate (44%). This may indicate that they do not need extensive stabilization, which is in line with a phase-based approach.

Contrary to what De Jongh and ten Broeke assume, none of the group therapists were replaced *during* the 20-week group intervention. However, many group therapists were comparatively inexperienced in conducting the treatment protocol, which may have resulted in an underestimation of the potential treatment effect.

There is a knowledge gap with respect to effective treatment of complex PTSD patients with PD, because this subgroup is rarely included in studies of exposure-based treatment. This limits the generalizability of previous research to this population which arguably is in greater need of treatment (Bradley, Greene, Russ, Dutra, & Westen, 2005). Furthermore, we found some evidence that affect management (“stabilization”) results in better outcomes than exposure treatment in complex PTSD, whether calculated in terms of drop-out, recovery, or improvement rates (meta-analysis, Dorrepaal et al., submitted). Interestingly, ten Broeke and de Jongh themselves included an important chapter on stabilization in their Handbook of EMDR where they advise on how to prepare PTSD patients for EMDR treatment (2013). To date, there is no outcome study directly comparing stabilization to EMDR in the treatment of child abuse related complex PTSD with severe PD. These treatments were compared in a small randomized controlled trial conducted by de Jongh and ten Broeke in a fairly complex patient population, but no assessment of child abuse, complex PTSD or PDs was reported (Ter Heide, Mooren, Kleijn, de Jongh, & Kleber, 2011). Based on their completers’ analysis of five patients per arm with a drop-out rate of 50%, they concluded that improvement on EMDR was small, but not worse than stabilization and that further comparison is needed.

The content of our time-limited “stabilizing” treatment protocol largely overlaps with other (effective) evidence-based stabilizing treatments (Cloitre et al., 2010; Zlotnick et al., 1997) and cognitive (processing) therapy (Resick et al., 2008) for PTSD, as well as with the recent use of dialectical behavior therapy for borderline PD with PTSD (Harned, Jackson, Comtois, & Linehan, 2010). This should encourage clinicians, as well as investigators, to offer their patients similar stabilization programs as ours, after which many patients are able to enter exposure-based treatments. Interventions that directly target general emotion-regulation skills enhance the effectiveness of psychotherapeutic cognitive (processing) therapy interventions (Berking et al., 2008).

In conclusion, the findings in our paper “Treatment compliance and effectiveness in complex PTSD patients with co-morbid personality disorder” (Dorrepaal et al., 2013) support stabilization in child abuse–related PTSD with PD, whereas our stabilizing treatment is not well suited to patients without PD. Although change score differences did not show a statistically significant superiority for stabilization, results of the secondary responders’ analyses were statistically significant and implied the clinical meaningfulness of combining skills training with cognitive therapy. Moreover, we demonstrated a very low drop-out rate in complex PTSD patients with PDs. Therefore, we recommend the use of stabilization treatment when PTSD patients drop out or cannot be included in evidence-based treatments like EMDR, prolonged exposure and cognitive (processing) therapy due to complexities such as severe personality disturbances. This conclusion is provisional on other treatments not having shown superior results in this population. We agree with de Jongh and ten Broeke that more research is needed with head-to-head comparisons in PTSD samples, particularly those consisting of complex PTSD, poorly educated, non-white, unemployed, medicated, suicidal, and personality disturbed individuals who have not responded to previous treatment, like the patients in our study. This is essential before assuming the effectiveness of any treatments in this understudied subgroup.

*Ethy Dorrepaal* Department of Psychiatry VU University Medical Center Amsterdam, The Netherlands EMGO Institute VU University Medical Center Amsterdam, The Netherlands PsyQ, Parnassiagroep The Hague, The Netherlands Email: e.dorrepaal@psyq.nl *Kathleen Thomaes* GGZ inGeest Amsterdam, The Netherlands Department of Psychiatry VU University Medical Center Amsterdam, The Netherlands Email: k.thomaes@vumc.nl *Johannes H. Smit* GGZ inGeest Amsterdam, The Netherlands Department of Psychiatry VU University Medical Center Amsterdam, The Netherlands EMGO Institute VU University Medical Center Amsterdam, The Netherlands *Dick J. Veltman* GGZ inGeest Amsterdam, The Netherlands Department of Psychiatry VU University Medical Center Amsterdam, The Netherlands *Adriaan W. Hoogendoorn* GGZ inGeest Amsterdam, The Netherlands Department of Psychiatry VU University Medical Center Amsterdam, The Netherlands *Anton J. L. M. van Balkom* GGZ inGeest Amsterdam, The Netherlands Department of Psychiatry VU University Medical Center Amsterdam, The Netherlands EMGO Institute VU University Medical Center Amsterdam, The Netherlands *Nel Draijer* GGZ inGeest Amsterdam, The Netherlands Department of Psychiatry VU University Medical Center Amsterdam, The Netherlands EMGO Institute VU University Medical Center Amsterdam, The Netherlands
